# Meta-analysis of the effectiveness of ankle pump exercise combined with anticoagulant therapy for the prevention of post-operative lower extremity deep vein thrombosis

**DOI:** 10.3389/fneur.2026.1899435

**Published:** 2026-07-14

**Authors:** Taotao Ding, Yi Chen, Mingkui Huang, Huihui Tu, Liezhi Wang

**Affiliations:** 1 The First People's Hospital of Wenling, Wenling, Zhejiang, China; 2 Taizhou Central Hospital, Taizhou, Zhejiang, China

**Keywords:** ankle pump exercise, anticoagulant therapy, deep vein thrombosis, meta-analysis, postoperative venous thromboembolism, thromboprophylaxis

## Abstract

**Background:**

Post-operative deep vein thrombosis (DVT) remains a common and potentially serious perioperative complication. Although anticoagulant therapy is the cornerstone of thromboprophylaxis, its effectiveness in alleviating postoperative lower-limb venous stasis is limited. Ankle pump exercises enhance venous return from the lower extremities and may provide a complementary antithrombotic effect when combined with anticoagulant therapy. However, the efficacy of ankle pump exercises as an adjunct to anticoagulation for preventing postoperative DVT has not been comprehensively evaluated. Therefore, this study aimed to assess the effectiveness of ankle pump exercises combined with anticoagulant therapy in preventing postoperative lower-extremity DVT.

**Methods:**

A systematic search was conducted in PubMed, EMBASE, Web of Science, the Cochrane Library, EBSCO-CINAHL, CNKI, VIP Database, and Wanfang Database from database inception to May 14, 2026. Randomized controlled trials (RCTs) comparing “ankle pump exercise combined with anticoagulant therapy” vs. “anticoagulant therapy alone” for the prevention of postoperative DVT were included. The primary outcome was the incidence of postoperative DVT. STATA 17.0 was used for meta-analysis. Subgroup analyses were performed according to the type of ankle pump exercise. Risk of bias was assessed using the Cochrane RoB 2 tool, and the quality of evidence was evaluated using the GRADE framework.

**Results:**

A total of 16 RCTs involving 2,169 postoperative patients were included. Meta-analysis demonstrated that, compared with anticoagulant therapy alone, ankle pump exercise combined with anticoagulant therapy significantly reduced the risk of postoperative DVT (RR = 0.26, 95% CI: 0.16–0.45). Moderate heterogeneity was observed across studies (I^2^ = 60.9%). Subgroup analysis showed that active ankle pump exercise provided a statistically significant and relatively consistent effect (RR = 0.21, 95% CI: 0.14–0.34; I^2^ = 0.0%), whereas passive or device-assisted ankle pump exercise exhibited substantial heterogeneity. Sensitivity analyses suggested relative stability of the findings; however, funnel plot asymmetry and Egger's test suggested the potential presence of publication bias. According to the GRADE assessment, the overall quality of evidence was low.

**Conclusions:**

The addition of ankle pump exercises to routine anticoagulant therapy may further reduce the risk of postoperative lower-extremity DVT, with active ankle pump exercises demonstrating a relatively more consistent protective effect. This combined approach may simultaneously address venous stasis and hypercoagulability and appears to be clinically feasible in postoperative settings. However, the overall certainty of the current evidence is limited by methodological shortcomings of the included studies, between-study heterogeneity, and potential publication bias. Therefore, the clinical benefits of this strategy should be interpreted cautiously, and further high-quality randomized controlled trials are required to confirm its effectiveness.

## 1 Introduction

Postoperative deep vein thrombosis (DVT) is a common and potentially serious complication during the perioperative period (1). Among these, lower extremity DVT may lead to limb swelling, pain, venous dysfunction, and post-thrombotic syndrome, and can further progress to fatal PE, thereby increasing postoperative mortality, prolonging hospitalization, and imposing substantial healthcare burdens (2, 3). Surgical patients are frequently exposed to multiple thrombogenic factors, including surgical trauma, anesthesia-related effects, inflammatory stress responses, vascular endothelial injury, and postoperative immobilization, all of which contribute to a high-risk thrombotic state (4, 5). The risk of lower extremity DVT is particularly pronounced in patients undergoing major orthopedic procedures, cancer surgery, advanced-age surgery, or prolonged bed rest (6). Therefore, optimizing postoperative thromboprophylaxis strategies remains a critical component of perioperative management.

At present, pharmacological anticoagulation remains the cornerstone of postoperative DVT prevention. Low-molecular-weight heparin, unfractionated heparin, warfarin, and direct oral anticoagulants reduce thrombus formation by inhibiting the coagulation cascade and have been widely adopted for perioperative thromboprophylaxis (7–9). Nevertheless, anticoagulant therapy alone still has important limitations. Some patients continue to develop lower extremity DVT despite receiving standardized anticoagulation therapy (10), suggesting that postoperative thrombosis is not solely driven by hypercoagulability. Moreover, intensified anticoagulation may increase the risks of bleeding, wound exudation, hematoma formation, and secondary intervention (11). Consequently, combining pharmacological anticoagulation with safe, simple, and reproducible non-pharmacological interventions may further improve postoperative thromboprophylactic efficacy.

According to Virchow's triad, venous stasis, hypercoagulability, and endothelial injury collectively contribute to the development of DVT (12). Anticoagulant agents primarily target the coagulation system but have limited effects on postoperative venous stasis caused by prolonged immobilization, impaired lower-limb muscle pump function, and reduced venous return (13). Ankle pump exercise, characterized by repetitive ankle dorsiflexion, plantar flexion, and rotational movements, is an active or passive lower-limb exercise modality capable of activating the gastrocnemius–soleus muscle pump, enhancing venous return, increasing venous blood flow velocity, and reducing local blood stasis (14–16). Therefore, ankle pump exercise and anticoagulant therapy may exert complementary antithrombotic effects through distinct mechanisms: the former primarily alleviates venous stasis, whereas the latter mainly suppresses hypercoagulability. Their combination may consequently provide more comprehensive thromboprophylactic benefits than anticoagulant therapy alone.

In recent years, several randomized controlled trials (RCTs) have investigated the efficacy of ankle pump exercise combined with anticoagulant therapy in preventing postoperative lower extremity DVT. However, substantial heterogeneity exists across studies regarding surgical type, baseline patient risk, implementation protocols for ankle pump exercise, types of anticoagulants, timing of intervention initiation, intervention duration, and follow-up endpoints. In addition, some studies were limited by relatively small sample sizes and insufficient methodological reporting, thereby restricting the robustness and generalizability of individual findings. Therefore, the present study conducted a systematic review and meta-analysis of available RCT evidence to evaluate the effectiveness of ankle pump exercise combined with anticoagulant therapy compared with anticoagulant therapy alone for the prevention of postoperative lower extremity DVT. Furthermore, subgroup and sensitivity analyses were performed to explore the influence of different ankle pump exercise modalities on pooled outcomes and study heterogeneity, with the aim of providing evidence-based support for optimizing comprehensive perioperative thromboprophylaxis strategies.

## 2 Methods

This systematic review was conducted in accordance with the Preferred Reporting Items for Systematic Reviews and Meta-Analyses (PRISMA) statement (17). The study protocol was prospectively registered in the International Prospective Register of Systematic Reviews (PROSPERO) (https://www.crd.york.ac.uk/prospero/; registration number: CRD420261398830).

### 2.1 Search strategy

A comprehensive literature search was performed in PubMed, the Cochrane Library, EMBASE, Web of Science, EBSCO-CINAHL, China National Knowledge Infrastructure (CNKI), VIP Database, and Wanfang Database from database inception to May 14, 2026. Randomized controlled trials (RCTs) investigating ankle pump exercise combined with anticoagulant therapy for the prevention of postoperative DVT were identified.

Both Medical Subject Headings (MeSH) and free-text terms were used, and the search strategies were adapted according to the characteristics of each database. In addition, the reference lists of included studies were manually screened to identify potentially relevant articles. The search terms included:

(ankle^*^ OR foot OR “lower limb” OR “lower extremity” OR “ankle pump exercise”) AND (pump OR exercise^*^ OR movement^*^ OR mobilization OR “range of motion” OR flexion OR dorsiflex^*^ OR plantarflex^*^ OR “ankle pump exercise”) AND (“deep vein thrombosis” OR DVT OR “deep venous thrombosis” OR “venous thromboembolism” OR VTE OR “venous thrombosis” OR “pulmonary embolism” OR PE OR thrombo^*^ OR embol^*^) AND (anticoagulant^*^ OR “anti-coagulant” OR heparin OR “low molecular weight heparin” OR LMWH OR enoxaparin OR dalteparin OR nadroparin OR warfarin OR rivaroxaban OR dabigatran OR apixaban OR edoxaban OR fondaparinux).

Although venous thromboembolism (VTE)-related terms were used to maximize search sensitivity, the outcome of interest was strictly limited to lower-extremity DVT.

The detailed search strategy is provided in Supplementary Material 2. EndNote 21 software was used for literature management, duplicate removal, and record screening.

Eligibility criteria were established according to the PICOS framework:

(1) Participants: Hospitalized patients aged ≥18 years undergoing any type of surgical procedure; (2) The intervention consisted of ankle pump exercises in addition to routine anticoagulant therapy, including both active ankle pump exercises and mechanically assisted ankle pump exercises; (3) Comparison: The same anticoagulant regimen as the intervention group without ankle pump exercise; (4) The primary outcome was the incidence of postoperative lower-extremity DVT; (5) Study design: Randomized controlled trials; (6) Language: English or Chinese.

The classifications of ankle pump exercises used in this study were defined as follows:

Active ankle pump exercise: Voluntary and conscious dorsiflexion, plantarflexion, and circumduction movements of the ankle performed by the patient. These movements activate the calf muscle pump, particularly the gastrocnemius–soleus complex, thereby enhancing venous return from the lower extremities.

Mechanically assisted ankle pump exercise: Interventions that utilize external mechanical devices to replicate the physiological effects of ankle pump movements. These include rhythmic ankle mobilization, intermittent plantar compression, or device-assisted activation of the calf muscle pump, all of which are intended to promote lower-extremity venous return.

The exclusion criteria were as follows:

(1) non-RCTs, including case-control studies, cohort studies, reviews, and case reports; (2) studies involving non-surgical populations or pediatric patients; (3) studies in which the intervention group received additional physical thromboprophylactic measures not matched in the control group; (4) duplicate publications, for which only the most complete or most recent version was included; (5) conference abstracts without accessible full texts or complete data; and (6) studies with incomplete or evidently erroneous data that could not be clarified after contacting the corresponding authors.

### 2.2 Data extraction

Literature screening and data extraction were independently conducted by two reviewers, and disagreements were resolved through discussion or consultation with a third reviewer.

Titles and abstracts were initially screened according to the eligibility criteria, followed by full-text assessment of potentially relevant studies. A predefined data extraction form was used to collect the following information: first author, publication year, country or region, sample size (intervention/control), age, sex distribution, type of surgery, intervention protocols in the experimental group, intervention duration and frequency, control group interventions, outcome data, and risk-of-bias assessment information.

When studies reported medians, interquartile ranges, or confidence intervals instead of means and standard deviations, data conversion was performed according to the recommendations of the Cochrane Handbook. If multiple time points were reported for the same outcome, data obtained immediately after the intervention were preferentially extracted. Missing data were requested from the corresponding authors whenever possible. Studies with unavailable or insufficient data after contact attempts were excluded, and only available data were analyzed.

### 2.3 Quality assessment

The methodological quality of included RCTs was assessed using the Cochrane Risk of Bias 2.0 (RoB 2.0) Excel tool. RoB 2.0 evaluates five domains: (1) bias arising from the randomization process; (2) bias due to deviations from intended interventions; (3) bias due to missing outcome data; (4) bias in outcome measurement; and (5) bias in the selection of reported results.

Based on responses to signaling questions within each domain, the overall risk of bias for each study was judged as “low risk,” “some concerns,” or “high risk.” Quality assessment was independently performed by two reviewers, and discrepancies were resolved through discussion or consultation with a third reviewer.

### 2.4 Statistical analysis

Meta-analysis was performed using Stata version 17.0. The incidence of postoperative DVT was used as the primary outcome measure. For dichotomous outcomes, risk ratios (RRs) with 95% confidence intervals (CIs) were calculated as pooled effect estimates. When event rates were low or zero-event studies were present, odds ratios (ORs) and the Peto OR method were applied where appropriate. For continuous outcomes, mean differences (MDs) with 95% CIs were used when measurement units were consistent across studies; otherwise, standardized mean differences (SMDs) with 95% CIs were calculated.

Statistical heterogeneity was assessed using Cochran's Q test and the I^2^ statistic. Heterogeneity was categorized as low (I^2^ < 25%), moderate (I^2^ = 25%−50%), or high (I^2^ > 50%) (18). Effect models were selected based on statistical, clinical, and methodological heterogeneity. A random-effects model was adopted when significant heterogeneity was detected (I^2^ > 50% or Q-test *p* < 0.10).

Sensitivity analyses were performed using a leave-one-out approach to evaluate the robustness of pooled estimates. When at least 10 studies were included for a given outcome, funnel plots and Egger's linear regression test were used to assess potential publication bias quantitatively.

### 2.5 Certainty of evidence assessment

The Grading of Recommendations Assessment, Development and Evaluation (GRADE) system was used to evaluate the certainty of evidence for each pooled outcome. Because all included studies were RCTs, the initial level of evidence was rated as “high.” The certainty of evidence was subsequently downgraded based on five domains: risk of bias, inconsistency, indirectness, imprecision, and publication bias. The final certainty of evidence was categorized as high, moderate, low, or very low.

## 3 Results

### 3.1 Literature search process and results

A total of 909 records were initially identified through searches of PubMed, the Cochrane Library, EMBASE, Web of Science, EBSCO-CINAHL, CNKI, VIP Database, and Wanfang Database.

**Figure 1:**
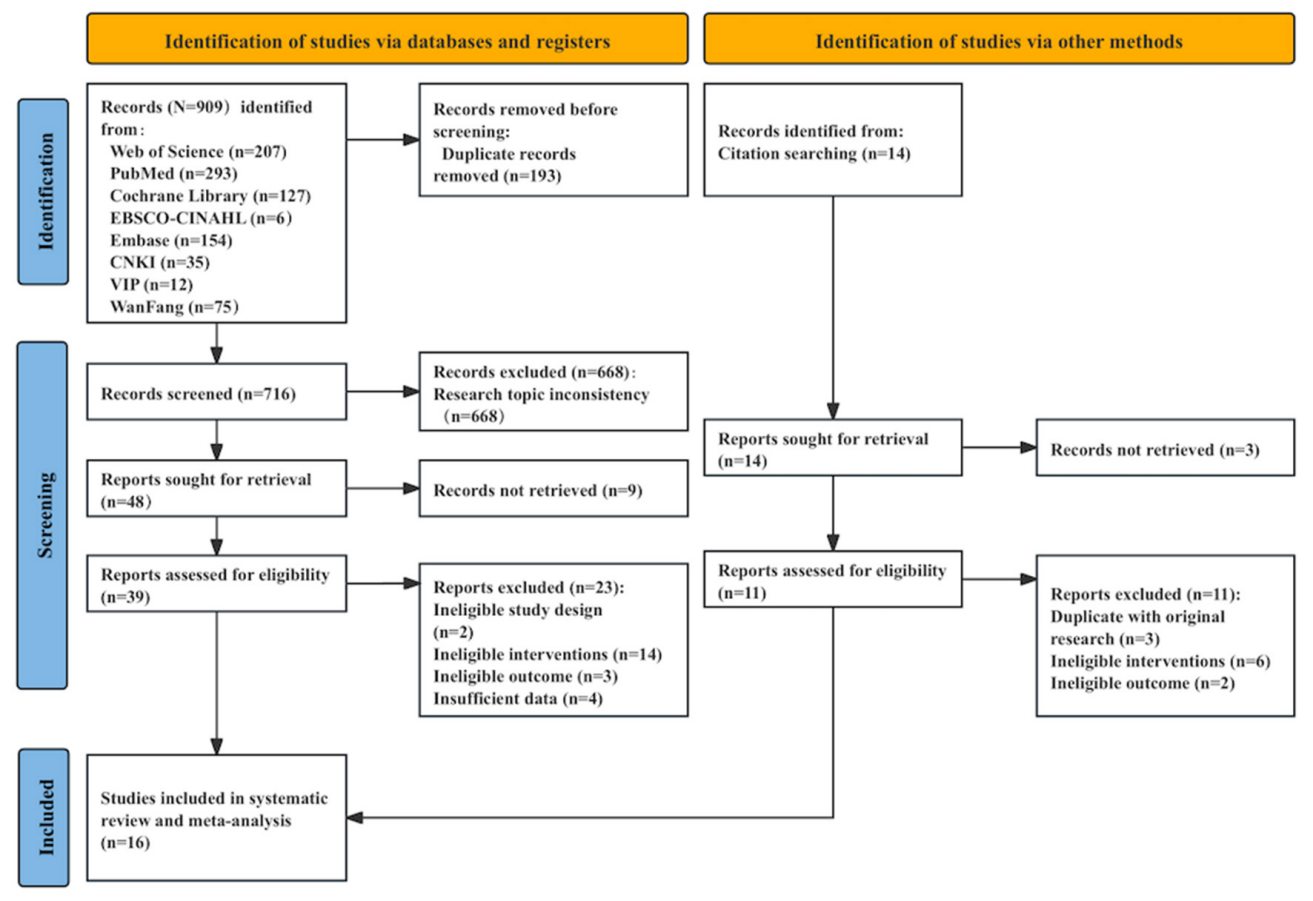
Flow chart of literature screening.

After the removal of duplicates, 716 records remained. Following title and abstract screening, 668 records that were not relevant to the study topic were excluded, leaving 48 articles for full-text assessment. Of these, 9 full-text articles could not be retrieved. After full-text review, 23 studies were excluded for the following reasons: non-randomized controlled trial design (*n* = 2), ineligible interventions (*n* = 14), incomplete data (*n* = 4), and incomplete outcome reporting (*n* = 3). Ultimately, 16 studies (19–34) met the inclusion criteria and were included in the meta-analysis. The study selection process is presented in Figure 1.

### 3.2 Characteristics of the included studies

A total of 16 randomized controlled trials published between 2005 and 2025 were included in this meta-analysis, involving studies conducted in five countries: China, Germany, Israel, Japan, and the United States. The total sample size was 2,169 postoperative patients, including 1,084 patients in the intervention group and 1,085 patients in the control group. The sample size of individual studies ranged from 45 to 260 participants.

The included populations comprised patients undergoing orthopedic surgery, gynecologic oncology surgery, cesarean section for preeclampsia, surgery for lower extremity varicose veins, and radical resection for hilar cholangiocarcinoma. Most studies primarily focused on the prevention of postoperative lower extremity deep vein thrombosis following major orthopedic procedures.

Regarding the interventions, all experimental groups received ankle pump exercises in combination with anticoagulant therapy. Among the included studies, 11 employed active ankle pump exercises, including standardized ankle pump exercises, breathing-guided ankle pump exercises, early postoperative ankle pump exercises, and active ankle dorsiflexion–plantarflexion movements. The remaining five studies utilized mechanically assisted ankle pump exercises, including continuous passive motion, foot pumps, plantar venous pulse systems, and intermittent pneumatic compression devices.

The concomitant anticoagulant regimens included unfractionated heparin, low-molecular-weight heparin, enoxaparin, rivaroxaban, aspirin, warfarin, and edoxaban. Control groups received anticoagulant therapy alone. The duration of intervention and follow-up varied considerably across studies, ranging from 7 postoperative days to 17 months. Specifically, two studies reported follow-up durations of 3 months, one study reported a follow-up duration of 17 months, whereas the remaining studies mainly focused on intervention or observation periods ranging from 7 days to 1 month after surgery. Detailed characteristics of the included studies are summarized in Table 1.

**Table 1:** Characteristics of included studies.

Study	Country	Study design	Population	Sample size (T)	Sample size (C)	Intervention (T)	Intervention (C)	Duration
Fuchs (19)	Germany	RCT	Post-operative orthopedic trauma patients	111	116	Arthroflow continuous passive ankle pump exercise & unfractionated heparin	Unfractionated heparin	3 months
Gelfer (20)	Israel	RCT	Post-operative total hip/knee arthroplasty patients	61	60	CECT mobile intermittent pneumatic compression system & aspirin	Aspirin	3 months
Sakai (21)	Japan	RCT	Primary total knee arthroplasty patients	58	62	A-V Impulse foot pump & edoxaban	Edoxaban	28 days postoperatively
Stannard (22)	USA	RCT	Blunt trauma/severe skeletal trauma patients	103	97	PlexiPulse foot pump & enoxaparin	Enoxaparin	17 months
Chen (23)	China	RCT	Post-operative lower extremity varicose vein patients	70	70	Standardized ankle pump exercise & oral rivaroxaban	Oral rivaroxaban	Postoperative to 14 days after discharge
Xia (24)	China	RCT	Post-operative hilar cholangiocarcinoma radical resection patients	57	57	Breathing-guided ankle pump exercise & low molecular weight heparin	Low molecular weight heparin	4 weeks postoperatively
Feng (25)	China	RCT	Post-operative orthopedic surgery patients	39	40	Breathing-guided active ankle dorsiflexion/plantarflexion exercise & low molecular weight heparin	Low molecular weight heparin	7 days postoperatively
Fang (26)	China	RCT	Post-operative gynecological tumor patients	23	22	Ankle pump exercise & low molecular weight heparin calcium	Low molecular weight heparin calcium	14 days postoperatively
Feng (27)	China	RCT	Elderly post-operative orthopedic surgery patients	53	53	Ankle pump exercise & low molecular weight heparin	Low molecular weight heparin	1 month postoperatively
Liu (28)	China	RCT	Post-operative lower extremity hip/knee surgery patients	91	91	Active ankle/foot exercise & low molecular weight heparin	Low molecular weight heparin	7 days postoperatively
Chen (32)	China	RCT	Pre-eclampsia cesarean section patients	50	50	Ankle pump exercise & low molecular weight heparin	Low molecular weight heparin	Postoperative
Pang (29)	China	RCT	Post-operative knee arthroplasty patients	43	42	Ankle pump training & low molecular weight heparin	Low molecular weight heparin	10 days
Tian (30)	China	RCT	Post-operative lower extremity joint replacement patients	56	56	Plantar venous pulse system & low molecular weight heparin calcium & aspirin & warfarin sodium	Low molecular weight heparin calcium & aspirin & warfarin sodium	≥14 days
Zhang (31)	China	RCT	Post-operative total hip arthroplasty patients	89	89	Early ankle pump exercise & low molecular weight heparin calcium	Low molecular weight heparin calcium	Not specified
Zhang (33)	China	RCT	Elderly post-operative hip fracture patients	50	50	low molecular weight heparin calcium & ankle pump exercise	low molecular weight heparin calcium	During hospitalization
Li (34)	China	RCT	Post-operative total knee surface replacement patients	130	130	Low molecular weight heparin calcium & active ankle/foot exercise	Low molecular weight heparin calcium	10 days

### 3.3 Quality assessment of the included studies

**Figure 2:**
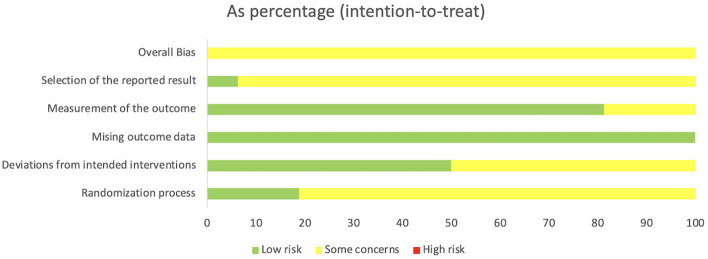
Overall distribution of risk of bias.

**Figure 3:**
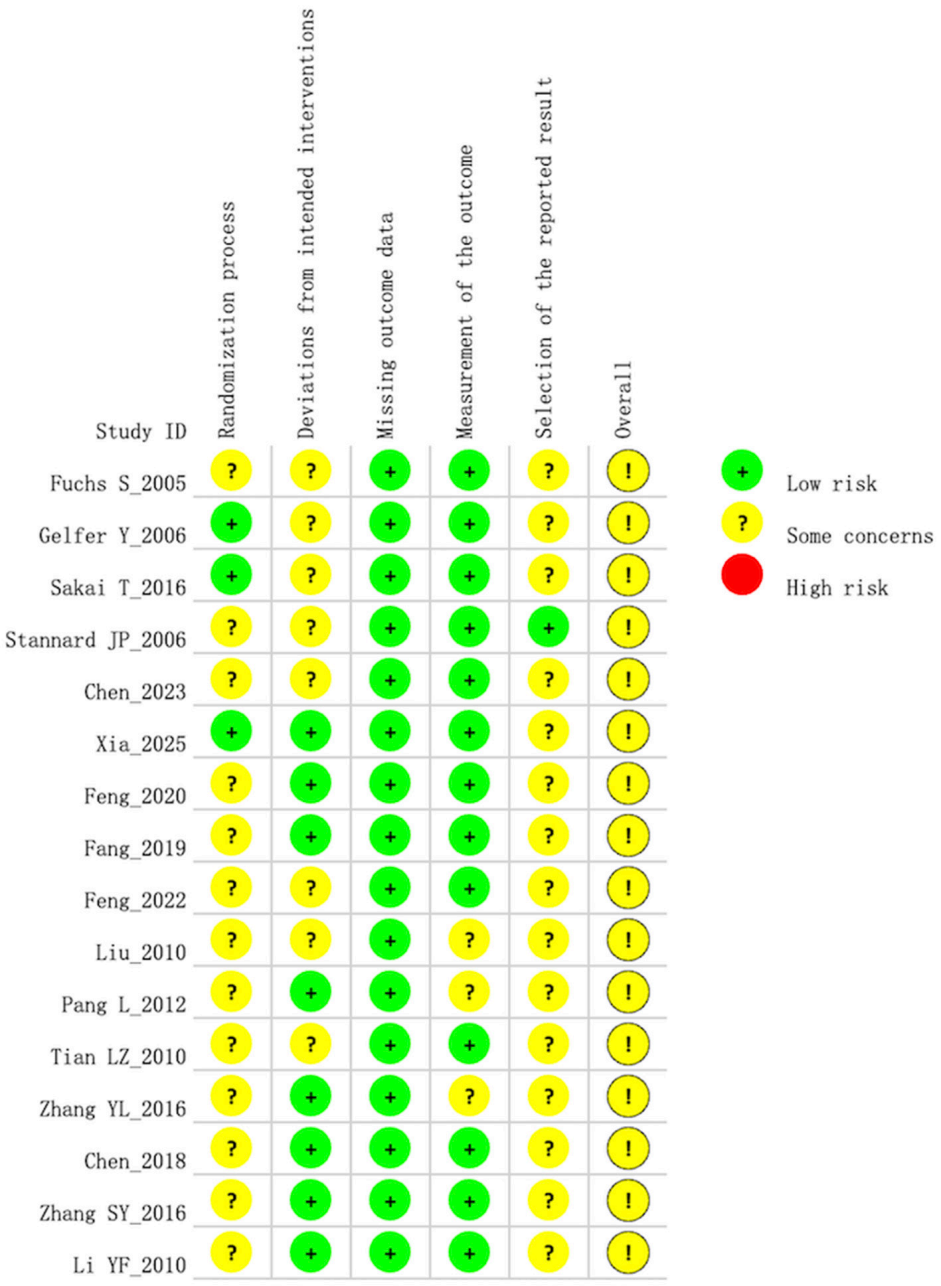
Characteristics of individual study bias risk.

The methodological quality of the 16 included randomized controlled trials was evaluated using the Cochrane Risk of Bias 2 (RoB 2) tool. Five domains were assessed: bias arising from the randomization process, bias due to deviations from intended interventions, bias due to missing outcome data, bias in outcome measurement, and bias in the selection of reported results. Each domain was rated as “low risk,” “some concerns,” or “high risk,” and an overall risk-of-bias judgment was subsequently assigned for each study. The detailed assessment results are presented in Figures 2, 3.

Based on the comprehensive evaluation across the five domains, all 16 studies (100%) were judged as having “some concerns” regarding overall risk of bias. No study was classified as having a high overall risk of bias, whereas none achieved a fully low-risk rating across all domains. These findings suggest that the overall methodological quality of the included studies was limited, primarily due to insufficient methodological reporting in earlier RCTs and the presence of potential sources of bias.

The major methodological concerns included: (1) lack of explicit reporting regarding blinding of outcome assessors or the use of objective outcome assessment criteria; (2) insufficient description of participant or investigator blinding procedures and adherence-monitoring strategies; (3) inadequate reporting of random sequence generation, allocation concealment methods, or baseline comparability assessments; and (4) absence of trial registration or prepublished statistical analysis protocols, making selective outcome reporting difficult to exclude.

### 3.4 Effect of ankle pump exercise combined with anticoagulant therapy on the incidence of postoperative lower extremity deep vein thrombosis

#### 3.4.1 Primary analysis

**Figure 4:**
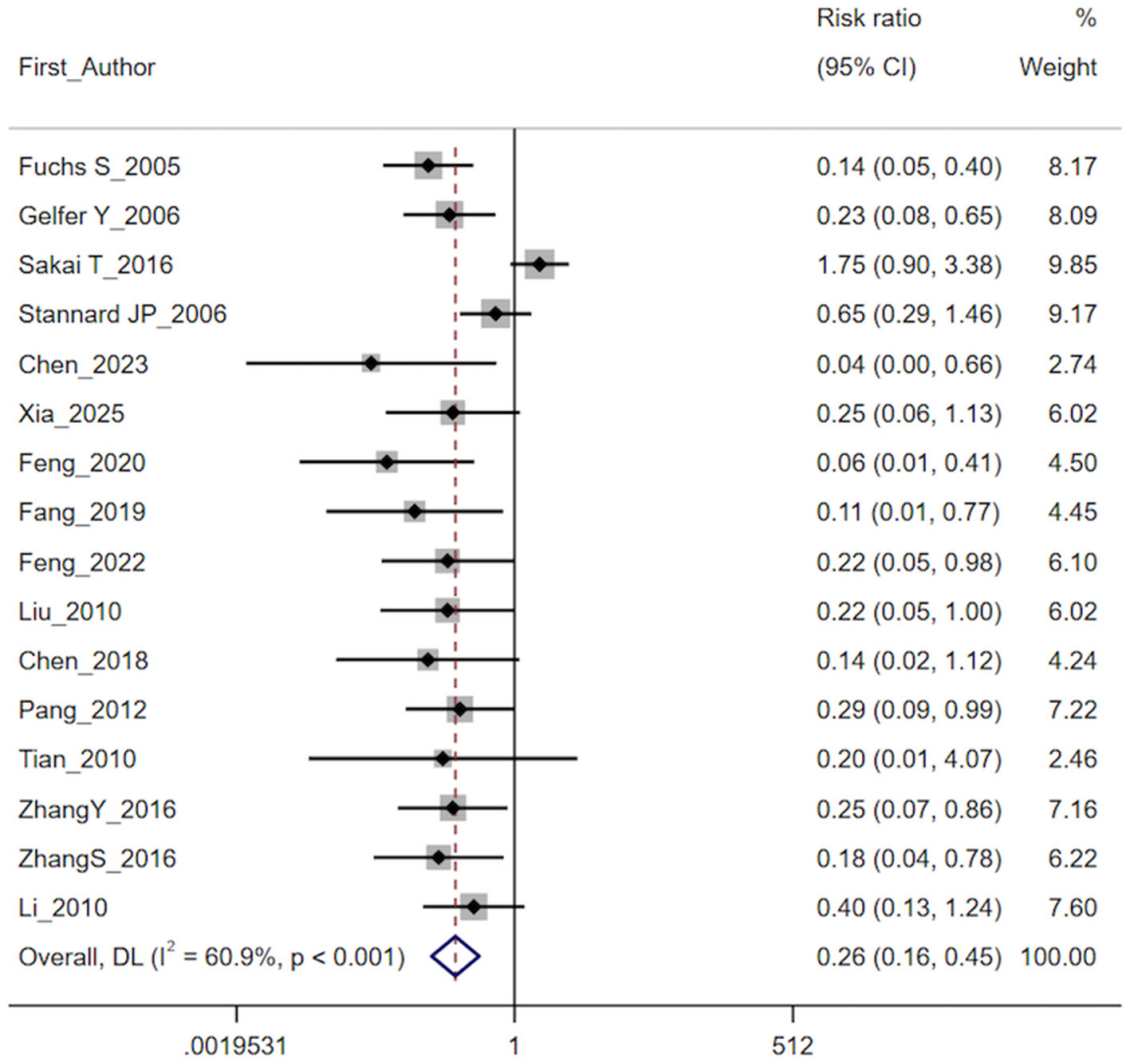
Forest plot of the effect of ankle pump exercise combined with anticoagulant therapy on the incidence of postoperative lower extremity deep vein thrombosis.

The incidence of postoperative DVT was defined as the primary outcome. A meta-analysis including 16 RCTs was performed using a random-effects model (DerSimonian–Laird method). As shown in Figure 4, compared with anticoagulant therapy alone, ankle pump exercise combined with anticoagulant therapy significantly reduced the incidence of postoperative DVT (RR = 0.26, 95% CI: 0.16–0.45). Significant heterogeneity was observed among the included studies (I^2^ = 60.9%, *p* < 0.001), meeting the predefined threshold for subgroup analysis.

#### 3.4.2 Subgroup analysis

To explore potential sources of heterogeneity and examine whether treatment effects differed according to the type of ankle pump intervention, subgroup analyses were performed based on the mode of ankle pump exercise: (1) active ankle pump exercise and (2) mechanically assisted ankle pump exercise.

**Figure 5:**
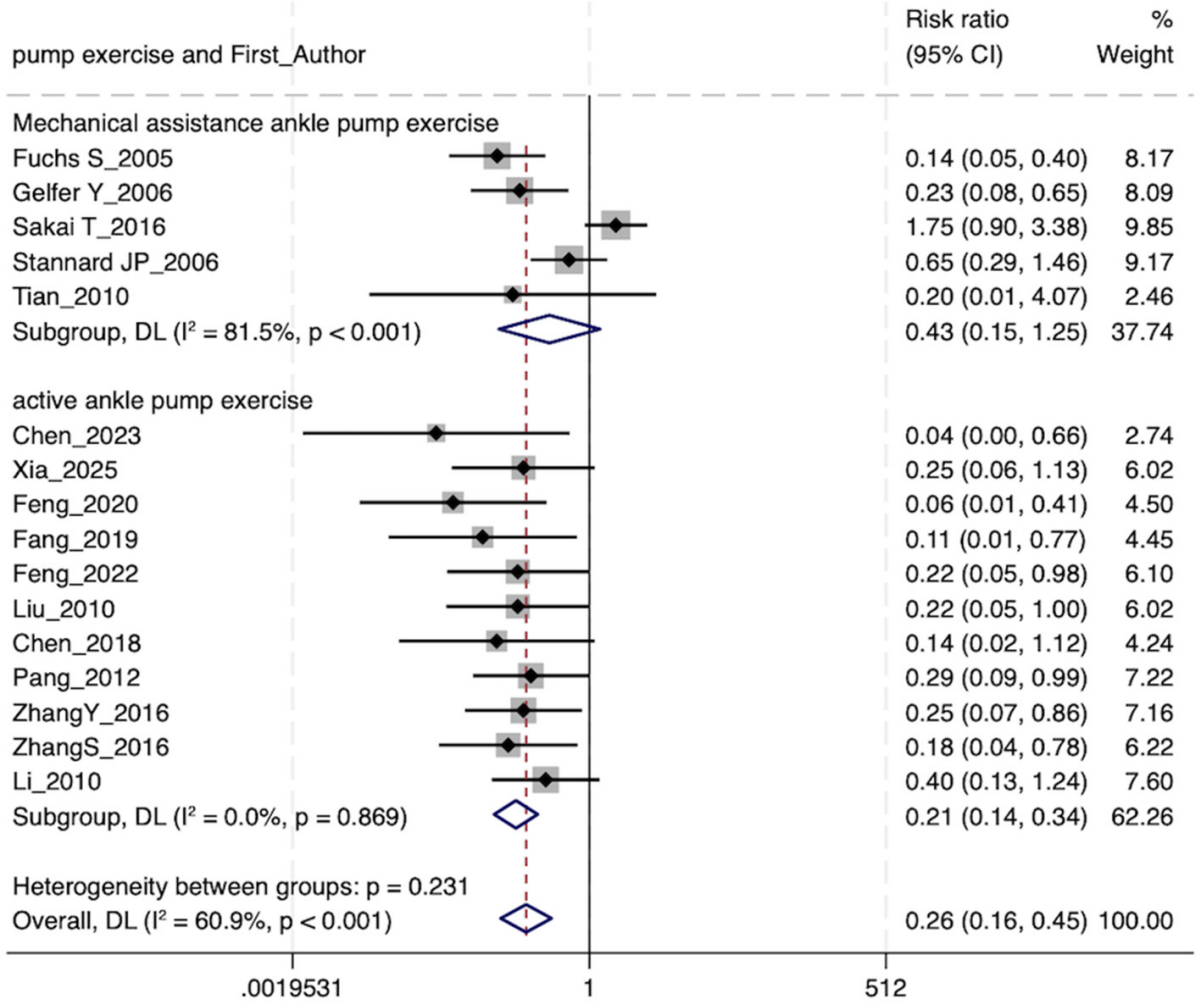
Subgroup analysis by ankle pump exercise form (active vs. passive/mechanically assisted).

As shown in Figure 5, the active ankle pump exercise subgroup included 11 studies and demonstrated a significant reduction in the risk of postoperative DVT (RR = 0.21, 95% CI: 0.14–0.34, *p* < 0.001), with no evidence of heterogeneity (I^2^ = 0.0%, *p* = 0.869). In contrast, the mechanically assisted ankle pump exercise subgroup included 5 studies and showed a non-significant pooled effect (RR = 0.43, 95% CI: 0.15–1.25, *p* = 0.125), accompanied by substantial heterogeneity (I^2^ = 81.5%, *p* < 0.001). The test for subgroup differences indicated no statistically significant difference between active and mechanically assisted ankle pump exercises (*p* = 0.231).

Given the substantial heterogeneity and non-significant pooled effect observed in the mechanically assisted ankle pump exercise subgroup, a leave-one-out sensitivity analysis was performed to identify potential sources of heterogeneity (Supplementary Material 4). Exclusion of the studies by Fuchs (19), Gelfer (20), Stannard (22), or Tian (30) did not materially alter the pooled results, which remained non-significant (all *p* > 0.05). However, after exclusion of the study by Sakai (21), the pooled effect became statistically significant (RR = 0.29, 95% CI: 0.13–0.65, *p* < 0.01), while heterogeneity decreased from 81.5% to 48.9% (I^2^ = 48.9%, *p* = 0.118). These findings suggest that the study by Sakai (21) was a major contributor to the observed heterogeneity and had a substantial influence on the pooled estimate for the mechanically assisted ankle pump exercise subgroup. Following its exclusion, mechanically assisted ankle pump exercise was also associated with a significantly lower risk of postoperative DVT, showing a direction of effect consistent with that observed for active ankle pump exercise.

#### 3.4.3 Sensitivity analysis

Sensitivity analysis was performed by sequentially excluding each of the 16 included studies (Supplementary Material 4). The pooled RR values remained relatively stable, ranging from 0.21 to 0.31 after exclusion of any single study, and all corresponding 95% CIs remained below the line of no effect (RR = 1). The pooled effect estimates remained statistically significant across sensitivity analyses, and the results were not materially influenced by any single study.

In addition, given the considerable variation in follow-up duration across the included studies (ranging from 7 days to 17 months), a sensitivity analysis stratified by follow-up time was conducted (Supplementary Material 4). Studies were categorized into a short-term follow-up group ( ≤ 14 days; 9 studies) and an extended follow-up group (>14 days; 7 studies). When the analysis was restricted to studies with short-term follow-up, the pooled effect estimate remained significant (RR = 0.21, 95% CI: 0.13–0.35) and was consistent in direction and magnitude with the primary analysis. These findings suggest that variations in follow-up duration were unlikely to have materially influenced the overall pooled effect estimate.

#### 3.4.4 Publication bias assessment

Publication bias for the outcome of postoperative DVT incidence was assessed using funnel plot analysis (Supplementary Material 5). The funnel plot demonstrated apparent asymmetry, with smaller studies exhibiting larger standard errors predominantly distributed on the left side of the funnel, whereas the lower-right region appeared relatively sparse. These findings suggest the potential presence of publication bias, indicating that small studies with positive findings may have been more likely to be published. Furthermore, Egger's regression test yielded a statistically significant result (*p* < 0.001), further supporting the possibility of publication bias.

### 3.5 GRADE assessment of evidence certainty

The certainty of evidence for the outcome of postoperative DVT incidence was evaluated using the GRADE framework, and the results are summarized in Supplementary Material 6. A total of 16 studies involving 2,169 participants were included, comprising 1,084 patients in the intervention group and 1,085 patients in the control group. The initial level of evidence was rated as high because all included studies were RCTs. After downgrading across two domains, the final certainty of evidence was rated as low.

Specifically, the certainty of evidence was downgraded for serious risk of bias due to methodological limitations in the included studies, particularly the lack of blinding procedures. In addition, publication bias was judged as serious because of the evident asymmetry observed in the funnel plot and the statistically significant Egger's test result (*p* < 0.001), suggesting that small studies with positive findings were more likely to be published. No downgrading was applied for inconsistency, indirectness, or imprecision.

## 4 Discussion

### 4.1 Principal findings of the present study

Based on 16 randomized controlled trials involving a total of 2,169 postoperative patients, this study systematically evaluated the effectiveness of ankle pump exercise combined with anticoagulant therapy for the prevention of post-operative lower extremity DVT. The meta-analysis demonstrated that, compared with anticoagulant therapy alone, the addition of ankle pump exercise significantly reduced the risk of postoperative DVT. Moreover, sensitivity analyses yielded stable results, suggesting a generally consistent direction of effect.

Further subgroup analyses demonstrated a consistent and significant protective effect of active ankle pump exercise. Although the initial pooled estimate for mechanically assisted ankle pump exercise did not reach statistical significance, sensitivity analyses suggested a potential benefit after exclusion of the study contributing most substantially to heterogeneity. Taken together, these findings indicate that the addition of ankle pump exercise to routine anticoagulant therapy may provide additional protection against post-operative DVT and could represent a useful adjunctive strategy for optimizing perioperative thromboprophylaxis.

### 4.2 Potential mechanisms underlying the combined effects of ankle pump exercise and anticoagulant therapy

The development of postoperative lower extremity DVT is not solely attributable to activation of the coagulation system, but rather results from the combined effects of altered hemodynamics, hypercoagulability, endothelial injury, and local inflammatory responses. Following surgery, tissue injury and inflammatory stress activate the coagulation cascade, placing patients in a hypercoagulable state (35). In addition, anesthesia, postoperative pain, limb immobilization, and prolonged bed rest may impair the function of the lower-extremity muscle pump, resulting in reduced venous return velocity and prolonged blood stasis within the venous system (36).

The primary physiological effect of ankle pump exercise is to restore and enhance calf muscle pump function (37). Repetitive ankle dorsiflexion, plantarflexion, and circumduction induce rhythmic contractions of the gastrocnemius, soleus, and intrinsic foot muscles, resulting in intermittent compression of the deep veins of the lower leg and facilitating venous return from the distal to the proximal circulation (37). In addition, the unidirectional action of venous valves helps prevent retrograde blood flow, enabling ankle pump exercise to function as a peripheral venous pumping mechanism that promotes venous drainage (38).

From the perspective of combined thromboprophylaxis, ankle pump exercise and anticoagulant therapy may exert complementary effects through distinct mechanisms of action. Anticoagulant agents primarily target the coagulation cascade by reducing thrombin generation and fibrin formation (39, 40). In contrast, ankle pump exercise acts mainly by enhancing calf muscle pump activity, increasing venous blood flow velocity, and reducing venous stasis (14, 38). Thus, the two interventions may address different pathophysiological components involved in postoperative DVT development. Furthermore, ankle pump exercise does not increase drug exposure and is characterized by its low cost, non-invasive nature, and ease of repeated implementation, making it a potentially practical adjunct to pharmacological thromboprophylaxis.

### 4.3 Interpretation of subgroup findings and heterogeneity

The primary meta-analysis demonstrated moderate-to-substantial heterogeneity, suggesting variability among studies in terms of patient populations, intervention protocols, and implementation characteristics. Subgroup analyses showed that active ankle pump exercise was associated with a relatively consistent protective effect and minimal between-study heterogeneity, whereas substantial heterogeneity was observed in the mechanically assisted ankle pump exercise subgroup.

One possible explanation is that different modes of ankle pump intervention may vary in their ability to activate the calf muscle pump. Active ankle pump exercise relies on voluntary muscle contraction and may therefore achieve more effective stimulation of the calf musculature and venous return. In contrast, mechanically assisted interventions differ considerably with respect to device type, pressure settings, treatment frequency, and duration, resulting in greater variability in intervention delivery across studies and potentially contributing to the observed heterogeneity.

Another potential source of heterogeneity relates to the diversity of surgical populations included in this review. The included studies involved patients undergoing major orthopedic procedures, trauma surgery, gynecologic oncology surgery, cesarean section, and hepatobiliary tumor surgery. These populations differ substantially in baseline DVT risk, postoperative mobility, and thromboprophylaxis strategies, all of which may influence treatment effects. For example, patients undergoing major orthopedic surgery typically experience more pronounced lower-limb immobilization and venous stasis and may therefore derive greater benefit from ankle pump exercise. Given the considerable variation in surgical procedures, anticoagulant regimens, intervention duration, and follow-up endpoints across studies, the pooled estimates should be interpreted with caution.

It is also noteworthy that anticoagulant regimens differed geographically. In the studies conducted in China, low-molecular-weight heparin was the predominant pharmacological prophylaxis strategy, whereas a broader range of anticoagulant agents was used in international studies (Supplementary Figure 7). Such regional differences in thromboprophylaxis practices may have introduced additional clinical heterogeneity and should be considered when interpreting the pooled findings.

Within the mechanically assisted ankle pump exercise subgroup, the study by Sakai et al. (21) exerted a substantial influence on overall heterogeneity. Sensitivity analyses demonstrated that exclusion of this study markedly reduced heterogeneity and resulted in a statistically significant pooled effect estimate. These findings suggest that specific characteristics related to patient selection, device design, anticoagulant regimen, or outcome assessment may have contributed disproportionately to the overall subgroup results. However, given the limited number of studies available in this subgroup, these findings should be interpreted cautiously.

### 4.4 Clinical implications

As a simple, low-cost, and non-invasive intervention, ankle pump exercise appears to be readily implementable within routine perioperative care. For patients at increased risk of venous thromboembolism, including older adults, individuals requiring prolonged bed rest, and those undergoing major orthopedic surgery, ankle pump exercise may provide additional thromboprophylactic benefits without increasing the risk of drug-related adverse events. Moreover, ankle pump exercise can be easily incorporated into standardized postoperative nursing and rehabilitation protocols and may be initiated early at the bedside under the guidance of healthcare professionals.

Given its favorable safety profile, ease of implementation, and potential complementary effect to pharmacological prophylaxis, ankle pump exercise may represent a practical adjunctive strategy for postoperative DVT prevention. Recent evidence has further highlighted the clinical relevance of thrombus phenotype and individualized management strategies in orthopedic patients with deep vein thrombosis, emphasizing the heterogeneity of postoperative DVT presentation and its implications for prophylactic interventions (41). Nevertheless, the current evidence remains limited, and further high-quality randomized controlled trials are needed to determine the optimal exercise protocol, timing of initiation, frequency, and duration of intervention across different surgical populations.

### 4.5 Strengths and limitations

This study systematically synthesized the available randomized controlled trial evidence to evaluate the effectiveness of ankle pump exercise combined with anticoagulant therapy for the prevention of postoperative lower-extremity DVT. Subgroup analyses, sensitivity analyses, and GRADE assessment were further performed to support the interpretation of the robustness and certainty of the findings.

Nevertheless, several limitations should be acknowledged, and the results should be interpreted with caution. First, the overall sample size of the included studies was limited, and several trials had relatively small sample sizes, which may have introduced small-study effects. Second, most of the included studies were conducted in China, resulting in a geographically concentrated evidence base that may limit the generalizability of the findings. Third, differences across studies in surgical procedures, ankle pump exercise protocols, types of anticoagulant agents, intervention duration, and follow-up endpoints may have contributed to clinical heterogeneity. In particular, the substantial variation in follow-up duration may have introduced bias when pooling event rates.

Fourth, all 16 included studies were judged as having “some concerns” according to the RoB 2 assessment, and none were rated as having a low risk of bias. These methodological limitations were mainly attributable to insufficient reporting of random sequence generation and allocation concealment in earlier RCTs, lack of blinding or inadequate reporting of blinding procedures in most studies, and the absence of trial registration or pre-specified statistical analysis plans, which made it difficult to exclude the possibility of selective reporting. The overall methodological quality of the included studies therefore limited the credibility of the evidence and was one of the main reasons why the certainty of evidence was rated as low by GRADE. Future studies should strictly follow the CONSORT statement and improve methodological reporting.

Fifth, some studies did not adequately report the diagnostic methods for DVT, screening time points, distinction between symptomatic and asymptomatic events, or whether pulmonary embolism events were assessed. These reporting deficiencies limited further exploration of outcome-related heterogeneity. In addition, funnel plot asymmetry and Egger's test suggested potential publication bias, indicating that small studies with positive findings may have been more likely to be published. Therefore, the pooled effect estimate may have been overestimated, and the true effect may be more conservative. Further large-scale, multicenter, high-quality randomized controlled trials with standardized intervention protocols are needed to verify the actual benefits and optimal implementation strategy of ankle pump exercise combined with anticoagulant therapy across different postoperative populations.

## 5 Conclusions

This meta-analysis suggests that adding ankle pump exercise to routine anticoagulant therapy may further reduce the risk of postoperative lower-extremity DVT, and the overall findings were relatively stable. Active ankle pump exercise showed a more consistent protective effect, whereas mechanically assisted ankle pump exercise may also provide potential benefit, although the latter was associated with substantial heterogeneity because it included devices with different mechanisms of action.

As a low-cost, non-invasive, and easily implemented intervention, ankle pump exercise may have adjunctive preventive value for patients at high risk of DVT, such as those undergoing orthopedic surgery, older adults, and patients requiring prolonged bed rest. However, given the limited sample size, methodological limitations, between-study heterogeneity, and potential publication bias of the included studies, the current certainty of evidence remains limited. The clinical benefits of this combined strategy should therefore be interpreted cautiously. Future large-scale, multicenter, high-quality randomized controlled trials with standardized intervention protocols are needed to further clarify the effectiveness of ankle pump exercise combined with anticoagulant therapy in different postoperative populations.

## Data Availability

The original contributions presented in the study are included in the article/Supplementary material, further inquiries can be directed to the corresponding author.
