# Supplementary material for: Meta-analysis of the effectiveness of ankle pump exercise combined with anticoagulant therapy for the prevention of post-operative lower extremity deep vein thrombosis
Source: Front Neurol. 2026 Jul 14;17:1899435. doi: 10.3389/fneur.2026.1899435 (PMC13407648; doi:10.3389/fneur.2026.1899435)
Supplement: Supplementary file 1 [file Supplementary_File_1.docx]

**Supplementary Material**

Supplementary Material 1 PRISMA checklist

Supplementary Material 2 Search strategy

Supplementary Material 3 Sensitivity analyses were performed in passive subgroups

Supplementary Material 4 Sensitivity analysis

Supplementary Material 5 Bias of publication

Supplementary Material 6 GRADE evidence quality assessment

Supplementary Material 7 Distribution of anticoagulation regimens in the control group

| **Supplementary Material 1 PRISMA checklist** | | | |
| --- | --- | --- | --- |
| **Section** **and** **Topic** | **Item** **#** | **Checklist** **item** | **Location**  **where** **item** **is** **reported** |
| **TITLE** | | |  |
| Title | 1 | Identify the report as a systematic review. |  |
| **ABSTRACT** | | |  |
| Abstract | 2 | See the PRISMA 2020 for Abstracts checklist. |  |
| **INTRODUCTION** | | |  |
| Rationale | 3 | Describe the rationale for the review in the context of existing knowledge. |  |
| Objectives | 4 | Provide an explicit statement of the objective(s) or question(s) the review addresses. |  |
| **METHODS** | | |  |
| Eligibility criteria | 5 | Specify the inclusion and exclusion criteria for the review and how studies were grouped for the syntheses. |  |
| Information sources | 6 | Specify all databases, registers, websites, organisations, reference lists and other sources searched or consulted to identify studies. Specify the date when each source was last searched or consulted. |  |
| Search strategy | 7 | Present the full search strategies for all databases, registers and websites, including any filters and limits used. |  |
| Selection process | 8 | Specify the methods used to decide whether a study met the inclusion criteria of the review, including how many reviewers screened each record and each report retrieved, whether they worked independently, and if applicable, details of automation tools used in the process. |  |
| Data collection process | 9 | Specify the methods used to collect data from reports, including how many reviewers collected data from each report, whether they worked  independently, any processes for obtaining or confirming data from study investigators, and if applicable, details of automation tools used in the process. |  |
| Data items | 10a | List and define all outcomes for which data were sought. Specify whether all results that were compatible with each outcome domain in each study were sought (e.g. for all measures, time points, analyses), and if not, the methods used to decide which results to collect. |  |
|  | 10b | List and define all other variables for which data were sought (e.g. participant and intervention characteristics, funding sources). Describe any assumptions made about any missing or unclear information. |  |
| Study risk of bias assessment | 11 | Specify the methods used to assess risk of bias in the included studies, including details of the tool(s) used, how many reviewers assessed each study and whether they worked independently, and if applicable, details of automation tools used in the process. |  |
| Effect measures | 12 | Specify for each outcome the effect measure(s) (e.g. risk ratio, mean difference) used in the synthesis or presentation of results. |  |
| Synthesis methods | 13a | Describe the processes used to decide which studies were eligible for each synthesis (e.g. tabulating the study intervention characteristics and comparing against the planned groups for each synthesis (item #5)). |  |
|  | 13b | Describe any methods required to prepare the data for presentation or synthesis, such as handling of missing summary statistics, or data conversions. |  |
|  | 13c | Describe any methods used to tabulate or visually display results of individual studies and syntheses. |  |
|  | 13d | Describe any methods used to synthesize results and provide a rationale for the choice(s). If meta-analysis was performed, describe the model(s), method(s) to identify the presence and extent of statistical heterogeneity, and software package(s) used. |  |
|  | 13e | Describe any methods used to explore possible causes of heterogeneity among study results (e.g. subgroup analysis, meta-regression). |  |
|  | 13f | Describe any sensitivity analyses conducted to assess robustness of the synthesized results. |  |
| Reporting bias assessment | 14 | Describe any methods used to assess risk of bias due to missing results in a synthesis (arising from reporting biases). |  |
| Certainty  assessment | 15 | Describe any methods used to assess certainty (or confidence) in the body of evidence for an outcome. |  |
| **RESULTS** | | |  |
| Study selection | 16a | Describe the results of the search and selection process, from the number of records identified in the search to the number of studies included in the review, ideally using a flow diagram. |  |
|  | 16b | Cite studies that might appear to meet the inclusion criteria, but which were excluded, and explain why they were excluded. |  |
| Study  characteristics | 17 | Cite each included study and present its characteristics. |  |
| Risk of bias in studies | 18 | Present assessments of risk of bias for each included study. |  |
| Results of  individual studies | 19 | For all outcomes, present, for each study: (a) summary statistics for each group (where appropriate) and (b) an effect estimate and its precision (e.g. confidence/credible interval), ideally using structured tables or plots. |  |
| Results of syntheses | 20a | For each synthesis, briefly summarise the characteristics and risk of bias among contributing studies. |  |
|  | 20b | Present results of all statistical syntheses conducted. If meta-analysis was done, present for each the summary estimate and its precision (e.g. confidence/credible interval) and measures of statistical heterogeneity. If comparing groups, describe the direction of the effect. |  |
|  | 20c | Present results of all investigations of possible causes of heterogeneity among study results. |  |
|  | 20d | Present results of all sensitivity analyses conducted to assess the robustness of the synthesized results. |  |
| Reporting biases | 21 | Present assessments of risk of bias due to missing results (arising from reporting biases) for each synthesis assessed. |  |
| Certainty of evidence | 22 | Present assessments of certainty (or confidence) in the body of evidence for each outcome assessed. |  |
| **DISCUSSION** | | |  |
| Discussion | 23a | Provide a general interpretation of the results in the context of other evidence. |  |
|  | 23b | Discuss any limitations of the evidence included in the review. |  |
|  | 23c | Discuss any limitations of the review processes used. |  |
|  | 23d | Discuss implications of the results for practice, policy, and future research. |  |
| **OTHER** **INFORMATION** | | |  |
| Registration and protocol | 24a | Provide registration information for the review, including register name and registration number, or state that the review was not registered. |  |
|  | 24b | Indicate where the review protocol can be accessed, or state that a protocol was not prepared. |  |
|  | 24c | Describe and explain any amendments to information provided at registration or in the protocol. |  |
| Support | 25 | Describe sources of financial or non-financial support for the review, and the role of the funders or sponsors in the review. |  |
| Competing interests | 26 | Declare any competing interests of review authors. |  |
| Availability of data, code and other materials | 27 | Report which of the following are publicly available and where they can be found: template data collection forms; data extracted from included studies; data used for all analyses; analytic code; any other materials used in the review. |  |

Supplementary Material 2 Search strategy

| Pubmed | | |
| --- | --- | --- |
| #1 | "Ankle Joint"[Mesh] OR "Ankle"[Mesh] OR "Foot"[Mesh] | 78411 |
| #2 | ((ankle*[tiab] OR foot[tiab] OR "lower limb"[tiab] OR "lower extremity"[tiab])  AND (pump[tiab] OR exercise*[tiab] OR movement*[tiab] OR mobilization[tiab] OR  "range of motion"[tiab] OR flexion[tiab] OR dorsiflex*[tiab] OR plantarflex*[tiab])) OR "Ankle pump exercise" | 49218 |
| #3 | "Deep Vein Thrombosis"[Mesh] OR "Venous Thromboembolism"[Mesh] OR "Venous Thrombosis"[Mesh] OR "Pulmonary Embolism"[Mesh] | 114434 |
| #4 | ("deep vein thrombosis"[tiab] OR DVT[tiab] OR "deep venous thrombosis"[tiab] OR  "venous thromboembolism"[tiab] OR VTE[tiab] OR "venous thrombosis"[tiab] OR  "pulmonary embolism"[tiab] OR PE[tiab] OR thrombo*[tiab] OR embol*[tiab]) | 672438 |
| #5 | "Anticoagulants"[Mesh] OR "Heparin"[Mesh] OR "Heparin, Low-Molecular-Weight"[Mesh] OR "Warfarin"[Mesh] OR "Rivaroxaban"[Mesh] OR "Dabigatran"[Mesh] | 156794 |
| #6 | (anticoagulant*[tiab] OR "anti-coagulant"[tiab] OR heparin[tiab] OR  "low molecular weight heparin"[tiab] OR LMWH[tiab] OR enoxaparin[tiab] OR  dalteparin[tiab] OR nadroparin[tiab] OR warfarin[tiab] OR rivaroxaban[tiab] OR  dabigatran[tiab] OR apixaban[tiab] OR edoxaban[tiab] OR fondaparinux[tiab]) | 188652 |
| #7 | (#1 OR #2) AND (#3 OR #4) AND (#5 OR #6) | 293 |
| Cochrane Library | | |
| #1 | (ankle* OR foot OR "lower limb" OR "lower extremity" OR "Ankle pump exercise"):ti,ab,kw | 58956 |
| #2 | (pump OR exercise* OR movement* OR mobilization OR "range of motion" OR flexion OR dorsiflex* OR plantarflex* OR "Ankle pump exercise"):ti,ab,kw | 287363 |
| #3 | #1 AND #2 | 23364 |
| #4 | ("deep vein thrombosis" OR DVT OR "deep venous thrombosis" OR "venous thromboembolism" OR VTE OR "venous thrombosis" OR "pulmonary embolism" OR PE OR thrombo* OR embol*):ti,ab,kw | 79395 |
| #5 | (anticoagulant* OR "anti-coagulant" OR heparin OR "low molecular weight heparin" OR LMWH OR enoxaparin OR dalteparin OR nadroparin OR warfarin OR rivaroxaban OR dabigatran OR apixaban OR edoxaban OR fondaparinux):ti,ab,kw | 29441 |
| #6 | #3 AND #4 AND #5 | 127 |
| EMBASE | | |
| #1 | 'ankle'/exp OR 'ankle pump exercise' | 162,019 |
| #2 | (ankle*:ti,ab OR foot:ti,ab OR 'lower limb':ti,ab OR 'lower extremity':ti,ab) AND (pump:ti,ab OR exercise*:ti,ab OR movement*:ti,ab OR mobilization:ti,ab OR 'range of motion':ti,ab OR flexion:ti,ab OR dorsiflex*:ti,ab OR plantarflex*:ti,ab) | 70,656 |
| #3 | 'deep vein thrombosis'/exp OR 'venous thromboembolism'/exp OR 'venous thrombosis'/exp OR 'pulmonary embolism'/exp | 336,204 |
| #4 | 'deep vein thrombosis':ti,ab OR dvt:ti,ab OR 'deep venous thrombosis':ti,ab OR 'venous thromboembolism':ti,ab OR vte:ti,ab OR 'venous thrombosis':ti,ab OR 'pulmonary embolism':ti,ab OR pe:ti,ab OR thrombo*:ti,ab OR embol*:ti,ab | 1,028,590 |
| #5 | 'anticoagulant agent'/exp OR 'heparin'/exp OR 'low molecular weight heparin'/exp | 942,387 |
| #6 | anticoagulant*:ti,ab OR 'anti-coagulant':ti,ab OR heparin:ti,ab OR 'low molecular weight heparin':ti,ab OR lmwh:ti,ab OR enoxaparin:ti,ab OR dalteparin:ti,ab OR nadroparin:ti,ab OR warfarin:ti,ab OR rivaroxaban:ti,ab OR dabigatran:ti,ab OR apixaban:ti,ab OR edoxaban:ti,ab OR fondaparinux:ti,ab | 291,685 |
| #7 | #1 OR #2 | 198,116 |
| #8 | #3 OR #4 | 1,135,156 |
| #9 | #5 OR #6 | 992,565 |
| #10 | #7 AND #8 AND #9 | 2,210 |
|  | RCT-154 |  |
| Web of Science | | |
| #1 | TS=(ankle* OR foot OR "lower limb" OR "lower extremity" OR "ankle pump exercise")  AND TS=(pump OR exercise* OR movement* OR mobilization OR "range of motion" OR  flexion OR dorsiflex* OR plantarflex* OR "ankle pump exercise") | 71,778 |
| #2 | TS=("deep vein thrombosis" OR DVT OR "deep venous thrombosis" OR  "venous thromboembolism" OR VTE OR "venous thrombosis" OR  "pulmonary embolism" OR PE OR thrombo* OR embol*) | 729,338 |
| #3 | TS=(anticoagulant* OR "anti-coagulant" OR heparin OR "low molecular weight heparin" OR  LMWH OR enoxaparin OR dalteparin OR nadroparin OR warfarin OR rivaroxaban OR  dabigatran OR apixaban OR edoxaban OR fondaparinux) | 234,376 |
| #4 | #3 AND #2 AND #1 | 207 |
| EBSCO (CINAHL) | | |
| #1 | SU (ankle* OR foot OR "lower limb" OR "lower extremity" OR "ankle pump exercise") AND SU (pump OR exercise* OR movement* OR mobilization OR "range of motion" OR flexion OR dorsiflex* OR plantarflex* OR "ankle pump exercise") | 12,574 |
| #2 | SU ("deep vein thrombosis" OR DVT OR "deep venous thrombosis" OR "venous thromboembolism" OR VTE OR "venous thrombosis" OR "pulmonary embolism" OR PE OR thrombo* OR embol*) | 81,464 |
| #3 | SU (anticoagulant* OR "anti-coagulant" OR heparin OR "low molecular weight heparin" OR LMWH OR enoxaparin OR dalteparin OR nadroparin OR warfarin OR rivaroxaban OR dabigatran OR apixaban OR edoxaban OR fondaparinux) | 35,418 |
| #4 | #1 AND #2 AND #3 | 6 |
| CNKI / VIP/ WANFANG | | |
| #1 | 踝泵运动 + 踝关节运动 + 足泵运动 + 踝泵 + 踝关节泵 |  |
| #2 | 深静脉血栓 + 下肢深静脉血栓 + DVT + 静脉血栓栓塞 + VTE + 肺栓塞 + 血栓形成 + 血栓栓塞 |  |
| #3 | 抗凝 + 抗凝药物 + 低分子肝素 + 肝素 + 华法林 + 利伐沙班 + 达比加群 + 阿哌沙班 + 依度沙班 + 磺达肝癸钠 |  |
| #4 | #1 AND #2 AND #3 |  |
|  | CNKI-35, VIP-12, WANFANG-75 |  |

Supplementary Material 3 Sensitivity analyses were performed in mechanically-assisted subgroup


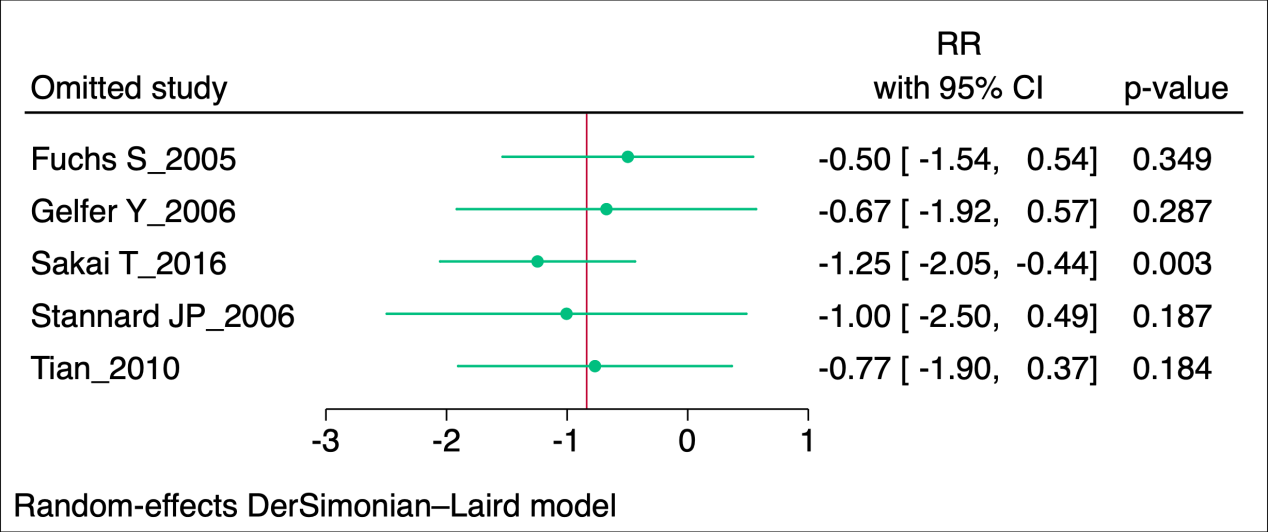


**Figure 1 Passive subgroup sensitivity analysis**


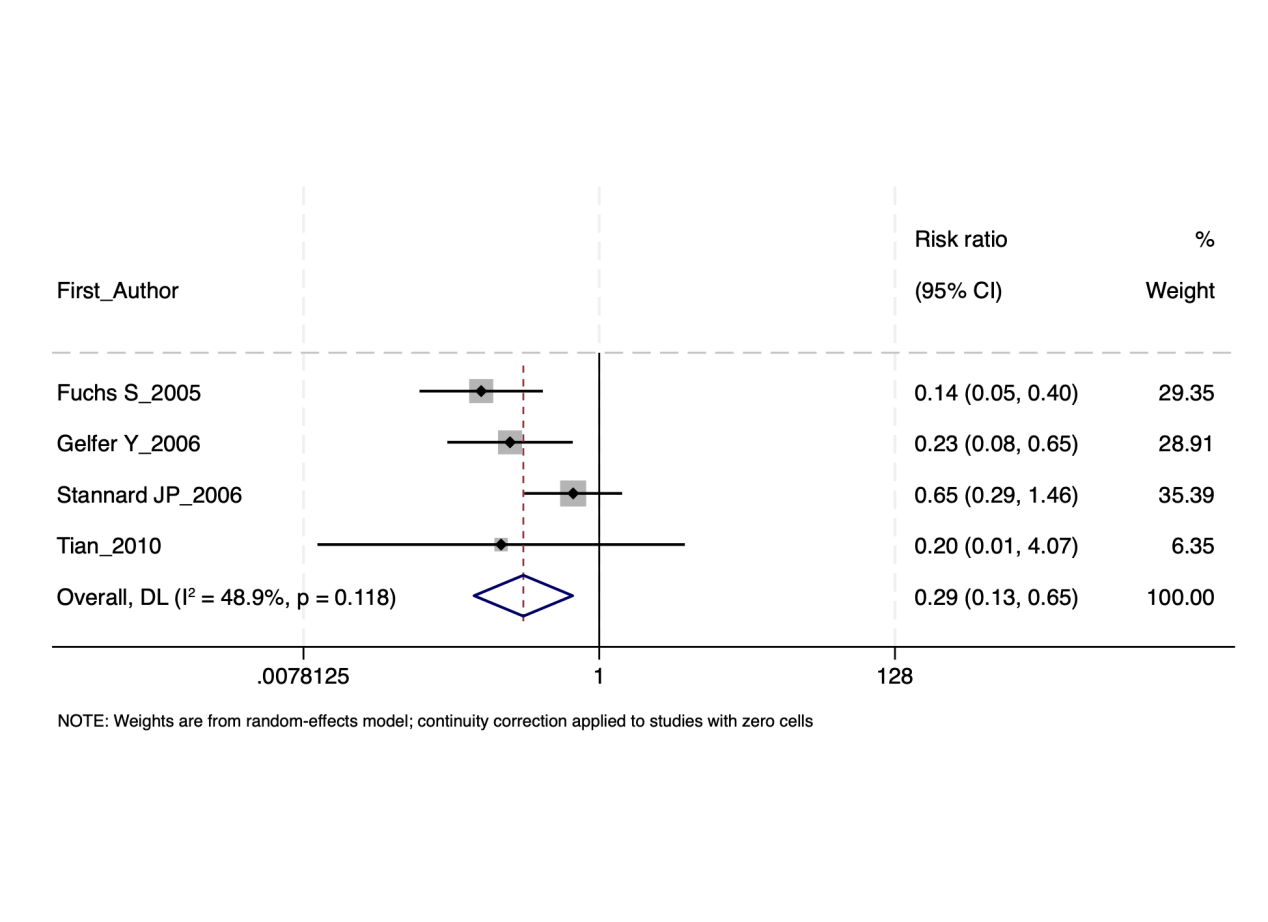


**Figure 2 Merger of the remaining four passive movement studies after removing Sakai T_2016**

Supplementary Material 4 Sensitivity analysis


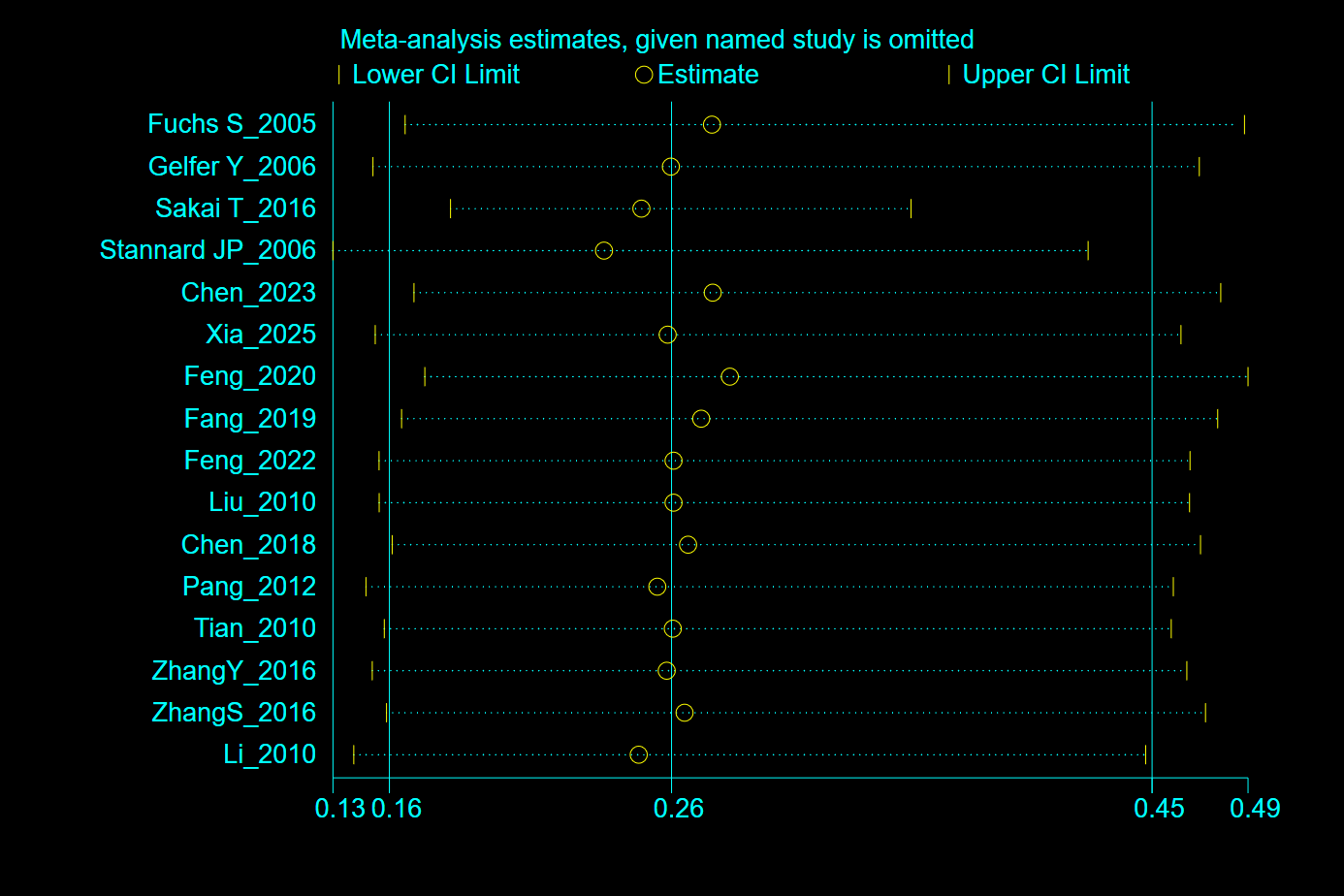


**Figure 3 Sensitivity analysis of the effect of ankle pump exercise combined with anticoagulant drugs on the incidence of postoperative lower extremity deep vein thrombosis**


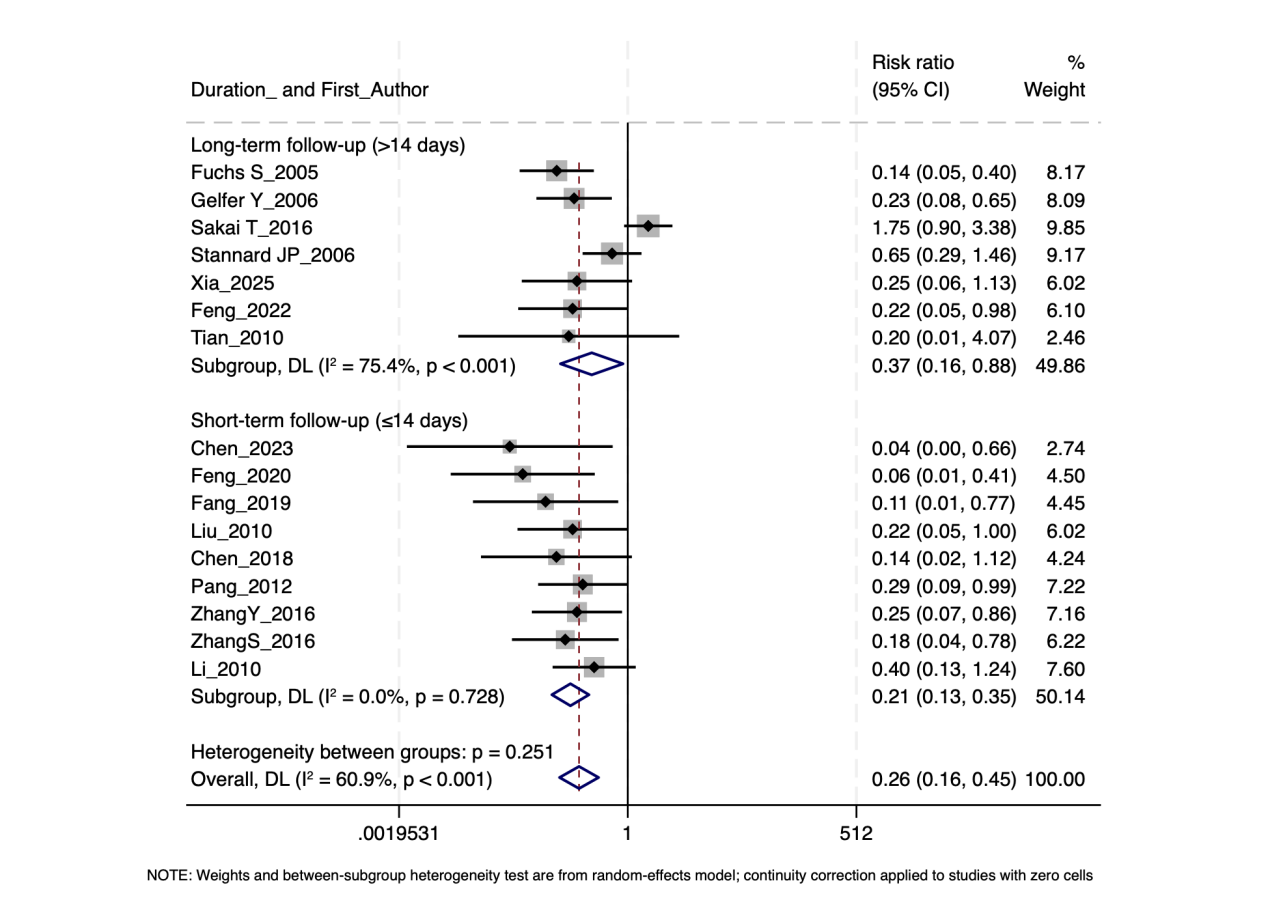


**Figure 4 Subgroup analyses by duration of follow-up**

**Supplementary Material 5 Publication bias assessment**


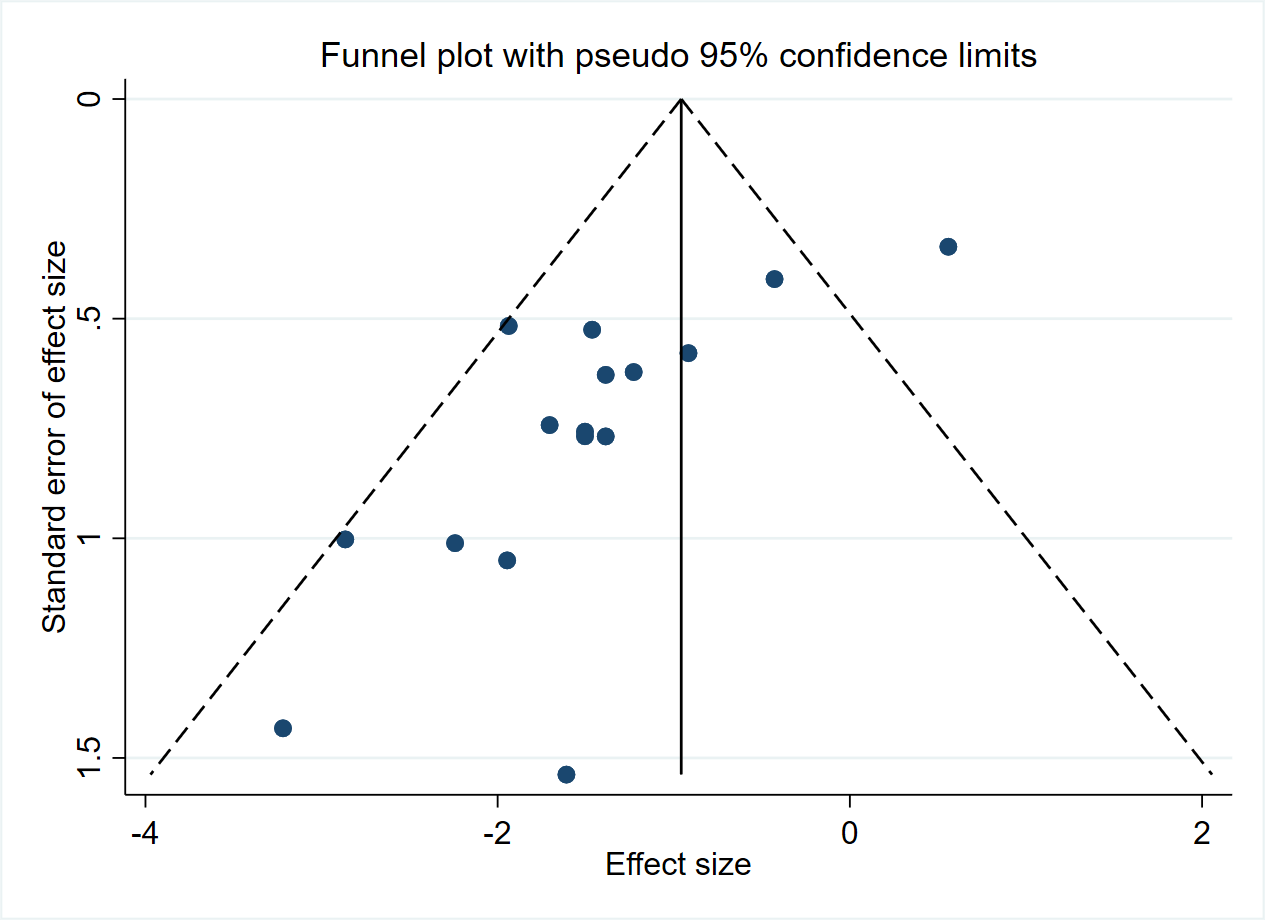


**Figure 5 Funnel plot of the effect of ankle pump exercise combined with anticoagulant drugs on the incidence of postoperative lower extremity deep vein thrombosis**


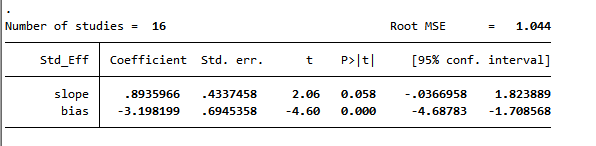


**Figure 6 Egger’s test result**

**Supplementary Material 6 GRADE evidence quality assessment**

Table 1 GRADE certainty assessment

| Outcome indicators | Number of  articles | Sample size | | Risk of bias | Inconsistency | Indirectness | Imprecision | Publication bias | RR 95% CI | Certainty |
| --- | --- | --- | --- | --- | --- | --- | --- | --- | --- | --- |
|  |  | Treatment group | Control group |  |  |  |  |  |  |  |
| Postoperative DVT | 16 | 1084 | 1085 | Serious^b^ | Not serious | Not serious | Not serious | Serious^I^ | **0.26(0.16-0.45)** | ⨁⨁◯◯ Low |

Footnotes:

a: Allocation concealment not performed;

b: Blinding not implemented;

c: Loss to follow-up not reported;

d: Intention-to-treat principle not properly considered;

e: Trial terminated early due to clear benefit;

f: Selective reporting of outcomes based on results;

G: Narrow or non-overlapping CI ranges, large I² value;

H: Small sample size, wide CI range;

I: High risk of publication bias

**Supplementary Material 7 Distribution of anticoagulation regimens in the control group**


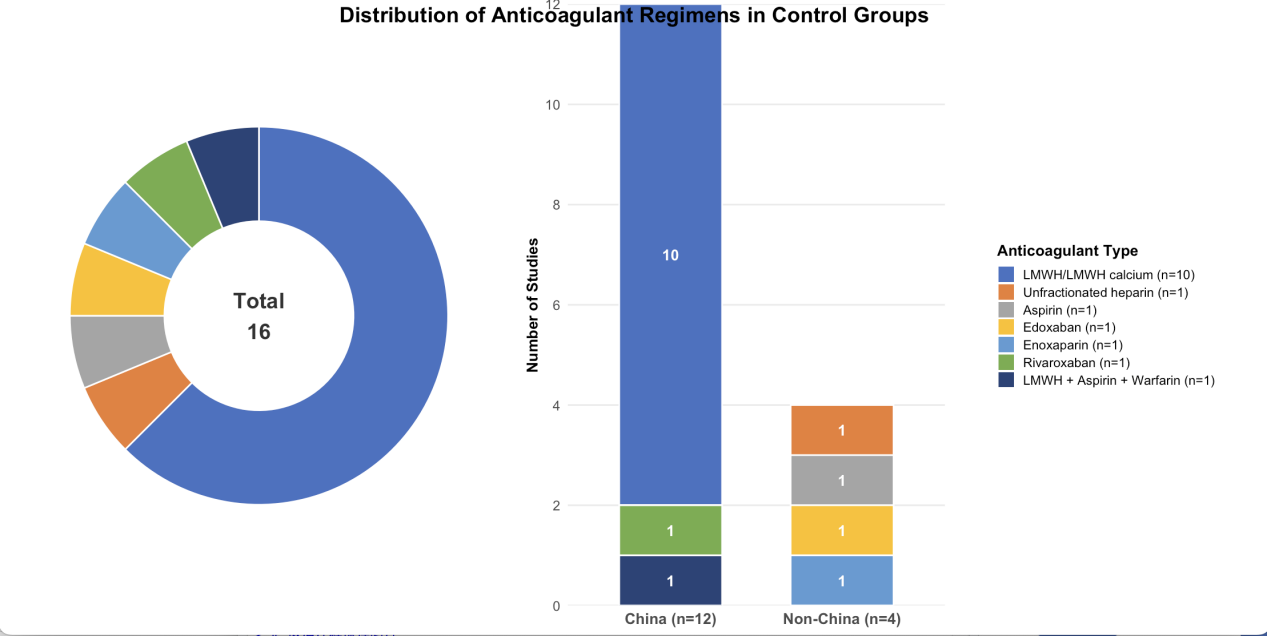


Figure 7. Distribution of anticoagulant regimens in control groups across the 16 included studies.
